# Ontology-based instance data validation for high-quality curated biological pathways

**DOI:** 10.1186/1471-2105-12-S1-S8

**Published:** 2011-02-15

**Authors:** Euna Jeong, Masao Nagasaki, Kazuko Ueno, Satoru Miyano

**Affiliations:** 1 Human Genome Center, Institute of Medical Science, University of Tokyo, Tokyo 108-8639, Japan

## Abstract

**Background:**

Modeling in systems biology is vital for understanding the complexity of biological systems across scales and predicting system-level behaviors. To obtain high-quality pathway databases, it is essential to improve the efficiency of model validation and model update based on appropriate feedback.

**Results:**

We have developed a new method to guide creating novel high-quality biological pathways, using a rule-based validation. Rules are defined to correct models against biological semantics and improve models for dynamic simulation. In this work, we have defined 40 rules which constrain event-specific participants and the related features and adding missing processes based on biological events. This approach is applied to data in Cell System Ontology which is a comprehensive ontology that represents complex biological pathways with dynamics and visualization. The experimental results show that the relatively simple rules can efficiently detect errors made during curation, such as misassignment and misuse of ontology concepts and terms in curated models.

**Conclusions:**

A new rule-based approach has been developed to facilitate model validation and model complementation. Our rule-based validation embedding biological semantics enables us to provide high-quality curated biological pathways. This approach can serve as a preprocessing step for model integration, exchange and extraction data, and simulation.

## Background

Modeling in systems biology is vital for the system-level understanding of biological processes and predicting the behavior of the system at each level. To obtain high-quality pathway databases, many important databases are built by manual curation sometimes with the aid of computer. A typical curation process is well illustrated in [1]. First, biological information resources are collected from literature, background knowledge, and other databases.

To create and evaluate pathway models, the information is organized into the building blocks in pathway databases. After creating the pathways models, the domain experts validate the created pathways and the curators update them based on appropriate feedback. This validation and update are an iterative procedure to obtain the desired specific annotated pathway.

Biological pathways are abstract representation of experimental data. Ontology-based representations for biological pathways have emerged because such formats provide the advantages of defining and constraining diverse data [2,3]. The pathway format is given in some representational language, while the generation of instance data is usually separated from ontology development. Although for the appropriate use of an ontology, formal definitions and informal documentation are given, it is sometimes difficult to avoid misassignment and misuse of ontology concepts. In the hierarchical structure of the ontology format, a more specific subclass should be selected instead of an upper class, such that a DNA binding process has at least one DNA as its participant. For the biological annotation, a suitable term should be selected from controlled vocabularies, such as cellular location for transcription. In addition, for dynamic models, more information which is usually not described in experimental data is required. Dimerization and polymerization need different stoichiometry coefficient. Likewise, there are important issues handled with care and they cannot be expressed formally in the ontology format. Based on this viewpoint, we are motivated to establish an ontology-based instance data validation tool.

Existing tools and inference engines [4-7] detect the misuse of features and check syntactic validation available in the ontology semantics. Ontology validation accomplishes generic ontology evaluation and debugging based on a schema and definitions for relationships in a conceptual model, such as logical consistency of the ontology, cardinality restriction, and subproperty axioms [8-10] On the other hand, there are some related works to complement knowledgebase by representing dynamics of the system, i.e., how to set relevant logical parameters for Petri net components [11,12], predicting operons and missing enzymes in metabolic databases [13]. In such works, the focus is given on representing dynamics of the system by adjusting initial values and parameters for components. Another important work is to verify pathway knowledgebase in terms of event relationships [14]. Racunas et al. in [14] carried out the verification on the level of the logical combinations of events, but without checking the biological meaning of individual events.

As a complement to such efforts, we had proposed a validation method to correctly represent biological semantics and system dynamics for biological pathways. [15]. On the basis of the previous work, we developed a rule-based approach for validating ontology-based instance data. As an ontology-based format, Cell System Ontology (CSO) [16] is used, which can represent biological pathways for simulation and visualization in OWL (Web Ontology Language) [17]. We have defined 40 rules embedding biological semantics to constrain event-specific participants with cardinality, participant types, cellular location, and others properties. In particular, 36 biological events are formalized on the basis of shared knowledge underlying biological pathways defined in CSO. We believe that our approach extends the expressiveness of the ontology and complements biological pathways with necessary properties, which aims to provide high-quality curated pathway models.

## Methods

We had defined three criteria for validating pathway models in terms of biological semantics and system dynamics as follows [15]:

**Criterion 1** A *structurally correct* model to be a bipartite graph with two disjoint sets.

**Criterion 2** A *biologically correct* model to represent the biological meaning of processes.

**Criterion 3** A *systematically correct* model to capture generic behaviors that govern the system dynamics.

For the three criteria, a rule-based approach is applied for validating biological pathways. A rule in this case is a form of reactive rules, i.e. event-condition-action rules. When the event happens, the corresponding condition is evaluated and the action is executed. Some rules are a form of condition-action rules that directly evaluate the specified condition with no event. That is, if the condition is satisfied, then the action is applied. Please note that the event part in reactive rules is different from biological events. Each rule specifies a variety of relationships on the basis of biological events, and consists of OWL constructors and axioms [17]. The available constructors and their correspondence with *SHOIQ* class expression [18] are summarized in Table 1. Each letter in *SHOIQ* indicates *S* for smallest propositionally closed description logic with transitive roles, *H* for role hierarchy, *O* for nominals, *I* for inverse roles, and *Q* for qualified number restrictions, respectively.

**Table 1:** OWL constructors and DL FOL equivalence.

Constructor	DL syntax	FOL syntax
intersectionOf	*C*_1_ ∩ ⋯ ∩ *C**_n_***	*C*_1_(*x*) Λ ⋯ Λ *C_n_*(*x*)
unionOf	*C*_1_ ∪ ⋯ ∪ *C_n_*	*C*_1_(*x*) V ⋯ V *C_n_*(*x*)
complementOf	¬ *C*	¬ C(*x*)
oneOf	{*a*_1_ ⋯ *a_n_*}	*x = a*_1_ V ⋯ V *x = a_n_*
allValuesFrom	∀*P.C*	∀*y*.(*P*(*x,y*) → *C*(*y*))
someValuesFrom	Ǝ*P.C*	Ǝ*y*.(*P*(*x, y*) Λ *C*(*y*))
minCardinality	*≥ nP.C*	Ǝ*^≥n^y*.(*P*(*x,y*) Λ *C*(*y*))
maxCardinality	≤ *nP.C*	Ǝ^≤^*^n^y*.(*P*(*x,y*) Λ *C*(*y*))

Relations used in rules are in typewriter type and the details are as follows: unary relations are classes; binary relations in all capital letters are properties; and pre-defined terms (instances) in CSO and variables for instances are in italics.

### Criterion 1: validation for structurally correct models

**Figure 1:**
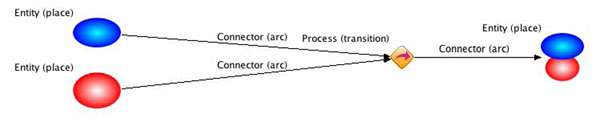
**Relationship between the CSO terms and the Petri net elements.** The CSO term is followed by the Petri net element in parentheses.

CSO uses an advanced Petri net named Hybrid Functional Petri net with an extension for the modeling and simulation of biological pathways [19]. In Petri nets, three elements, including place, transition, and arc, are defined. In order to be more intuitive for biological investigations, the Entity, Process, and Connector classes are used to denote place, transition, and arc element, respectively, in CSO. For the details of CSO and its schema, please refer to [16]. The relationship of the CSO classes and the Petri net elements is graphically summarized in Figure 1. The Entity class is used to represent objects, e.g. mRNA, protein, and small molecules. The Process class is used to represent biological events, e.g. phosphorylation, acetylation, and translocation. The relationship between Entity and Process is represented by the Connector class, i.e. indicating which entity is involved in a process.

In the Petri net architecture, an entity reflects the concentration of the substance and a process has a speed that depends on the concentration of the incoming entity. A connector transfers tokens from the input entity to the process or from the process to the output entity. Connector has several Petri nets related properties for simulation, such as initial value (concentration), minimum value, maximum value, and kinetics. Because of this reason, Connector is defined as a class in CSO. There are four types of connectors which imply the role of the involved entity in the process, including substrate, inhibitor, activator, and product.

**Figure 2:**
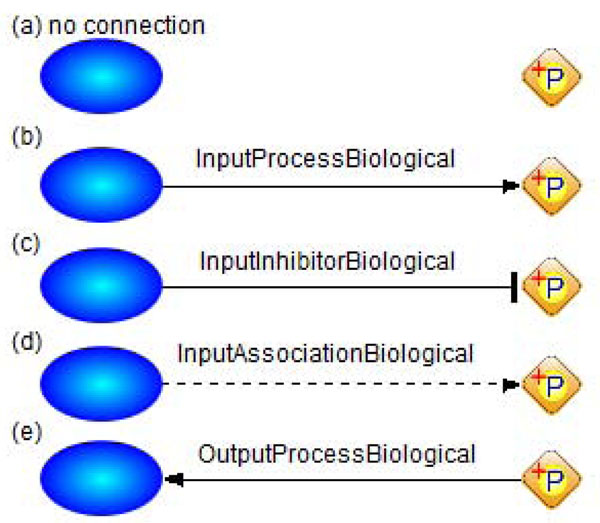
**Valid connections between Process and Entity via Connector.** Legend for icons on the left: blue ovals for Entity, diamond for Process, and lines between Process and Entity for Connector. Each connection shows the type of the connector: (a) no connection; (b) InputProcessBiological for substrate; (c) InputInhibitorBiological for inhibitor; (d) InputAssociationBiological for activator; and (e) OutputProcessBiological for product.

We define that one entity can participate in a process with only one role. In other words, more than two connectors associated with the same pair of an entity and a process are not allowed. The valid connections among Process, Entity, and Connector are five types as shown in Figure 2. Process can have multiple Connectors and each connector can have only one Entity by definition in CSO. To constrain the relation among three classes, we defined a rule for detecting any invalid connection in the below.

The condition part checks whether there exist more than two connectors for a given pair of process and entity. If the condition is true, then perform the action. This rule requires user intervention to select a correct connector because it is difficult to decide which connector is correct without understanding the details of the interaction [15].

In the rule description, *E*, *C*, and *A* denote *Event*, *Condition*, and *Action*, respectively.

Rule for valid connection

*E*: Process(*x*_1_) Λ Entity(*x*_2_)

*C*: ¬ [Ǝ^≤1^*x*_3_ CONNECTOR(*x*_1_,*x*_3_) Λ {InputProcessBiological(*x*_3_) V InputInhibitorBiological(*x*_3_) V InputAssociationBiological(*x*_3_) V OutputProcessBiological(*x*_3_)} Λ ENTITY(*x*_3_,*x*_2_)]

A: alert

### Criterion 2: validation for biologically correct models

Biological pathways consist of a series of interactions among entities. As described before, the Process class represents biological events each of which has characteristic features such as the type of molecules performing the event, the number of molecules involved, and the location which the event occurs. For example, autophosphorylation is a biological event to add a phosphate to a protein kinase by virtue of its own enzymic activity. Hence, autophosphorylation is different from phosphorylation because it occurs without any enzyme. Such definition is usually written in the natural language for the human users. To facilitate curation procedure, we defined four types of constraints for biological events which have specific requirements as follows:

**Cardinality constraint** A biological event needs constraints for the number of participating entities.

**Type constraint** A biological event needs a specific type of the entity, such as small molecule and DNA.

**Property constraint** An entity involved in a biological event needs to have a specific value for the property such as protein modification, cellular location, and stoichiometric coefficient.

**Property relationship constraint** For two entities involved in the same biological event, there needs a specific relationship between the values of the same property, such that two values should be same.

In this article, we have defined 36 rules for the 36 biological events. The 36 rules are divided into five groups depending on the necessary constraints for convenience. In the following rules, the action part will be different depending on the constraints in the condition part. Basically, the action is to show users an error message when the constraints are not satisfied. We use abbreviations as follows: hasInput(*p*_1_, *e*_1_) implies that a process *p*_1_ has an entity *e*_1_ which is connected to *p*_1_ via one of three input connectors InputAssociationBiological, InputInhibitorBiological, and InputProcessBiological; hasInputProcess(*p*_1_, *e*_1_) means that *e*_1_ is connected to *p*_1_ via InputProcessBiological; and hasOutput(*p*_1_, *e*_1_) means that *e*_1_ is connected to *p*_1_ via OutputProcessBiological. The types of connectors are already shown in Figure 2. For each *e*_1_*,* we called it as an input, inputprocess, output entity, respectively. In addition, for the pre-defined instances in CSO, the apostrophe prefix is used, such as *’FT_phosphorylated* and *’ME_Binding.* CSO provides pre-defined common vocabularies to annotate biological information. It allows to reuse existing structured information from other resources and to guide the allowable values for annotating biological information. Due to limitation of space, we list only several rules here. The formal description and full list of the rules are given in Additional file 1.

#### Group 1: rules that need cardinality and type constraints

Biological events in this group are required to have a specific type of an entity and/or a specific number of the entity. For example, DNA binding is defined as binding of a protein to the promoter/enhancer of a gene. The rule for DNA binding describes that there needs at least two more inputprocess entities; one of inputprocess entities has the type as Dna; and the product of DNA binding should have the type as Complex. Both Dna and Complex are subclasses of Entity in the hierarchy of CSO.

Rule for DNABinding

*E*: Process(*x*_1_) Λ BIOLOGICALEVENT(*x*_1_, *’ME_DNABinding*)

*C*: ¬ [Ǝ^≥2^*x*_2_,Ǝ^≥1^*x*_3_ hasInputProcess(*x*_1_,*x*_2_) such that for one of *x*_2_s, Dna(*x*_2_) Λ hasOutput(*x*_1_,*x*_3_) Λ Complex(*x*_3_)]

#### Group 2: rules that need cardinality and SEQUENCEFEATURE property constraints

This group includes rules for the sequence relevant interaction such as post-translational modification. In the rules, hasFeature(*x*_1_,’*x*_2_) means that an entity *x*_1_ has a feature type as ’*x*_2_ where ’*x*_2_ is a predefined term for FeatureType.

Rule for Acetylation

*E*: Process(*x*_1_) Λ BIOLOGICALEVENT(*x*_1_, *’ME_Acetylation*)

*C*: ¬ [Ǝ^=1^*x*_2_ , Ǝ*x*_3_, Ǝ*x*_4_, Ǝ*x*_5_ hasInputProcess(*x*_1_,*x*_2_) Λ hasOutput(*x*_1_ ,*x*_3_) Λ Entity(*x*_2_) Λ Entity(*x*_3_) Λ hasFeature(*x*_3_, ’*FT_Acetylated)* Λ UNIFICATIONXREF(*x*_2_,*x*_4_) Λ UNIFICATIONXREF(*x*_3_,*x*_5_) Λ sameAs(*x*_4_,*x*_5_)]

The acetylation event generates a chemically acetylated protein that has its FEATURETYPE as *’FT_Acetylated.* In the condition part, the external references for two entities, i.e, the values of UNIFICATIONXREF (*x*_4_ and *x*_5_) for the input *x*_2_ and output *x*_3_ entities, have to be the same because *x*_3_ is a modified form of *x*_2_

Rule for Autophosphorylation

*E*: Process(*x*_1_) Λ BIOLOGICALEVENT(*x*_1_,*’ME_Autophosphorylation*)

*C*: ¬ [Ǝ^=1^*x*_2_,Ǝ*x*_3_, Ǝ*x*_4_, Ǝ*x*_5_, Ǝ^=1^*x*_6_ hasInputProcess(*x*_1_,*x*_2_) Λ hasOutput*(x*_1_,*x*_3_) Λ Entity(*x*_2_) Λ Entity(*x*_3_) Λ FEATURETYPE(*x*_3_, *’FT_Phosphorylated*) Λ UNIFICATIONXREF(*x*_2_,*x*_4_) Λ UNIFICATIONXREF(*x*_3_,*x*_5_) Λ sameAs(*x*_4_,*x*_5_) Λ hasInput(*x*_1_,*x*_6_) Λ Entity(*x*_6_)]

For autophosphorylation, the condition part describes that the output entity is a phosphorylated form of the inputprocess entity when no enzyme is present. Two properties, hasInputProcess(*x*_1_,*x*_2_) and hasInput(*x*_1_,*x*_6_), imply that *x*_2_ and *x*_6_ are the same entity. The process has one output entity whose feature type is defined as *’FT_Phosphorylated*.

#### Group 3: cardinality and STOICHIOMETRY property constraints

There are three events that indicate the chemical union of identical molecules. Depending on the definition in CSO, the stoichiometric coefficient of an inputprocess entity is 2 for dimerization, more than 2 and less than 21 for oligomerization, and more than 20 for polymerization.

In the below, the rule describes that it needs one inputprocess entity whose stoichiometry coefficient is equal to 2 and one output entity whose type is Complex.

Rule for Dimerization

*E:* Process(*x*_1_) Λ BIOLOGICALEVENT(*x*_1_,*’ME_Dimerization*)

*C*: ¬ [Ǝ^=1^*x*_2_,Ǝ^=1^*x*_3_,Ǝ^=1^*x*_4_ hasInputProcess(*x*_1_,*x*_2_) Λ Entity(*x*_2_) Λ hasStoichiometry(*x*_2_,*x*_3_) Λ (*x*_3_ = 2) Λ hasOutput(*x*_1_,*x*_4_) Λ Complex(*x*_4_)]

#### Group 4: rules that need cardinality and CELLCOMPONENT property constraints

In some biological events, cellular location of participating entities is important. For example, the internalization and nuclear export events are the movement of the inputprocess entity from extracellular/plasma membrane to cytosol, and from nucleoplasm to cytoplasm, respectively, while the translocation event requires that the inputprocess and output entities just have different cellular locations.

Rule for Internalization

*E*: Process(*x*_1_) Λ BIOLOGICALEVENT(*x*_1_, *’ME_Internalization*)

*C*: ¬ [Ǝ^=1^*x*_2_, Ǝ^=1^*x*_3_, Ǝ*x*_4_, Ǝ*x*_5_ hasInputProcess (*x*_1_,*x*_2_) Λ Entity(*x*_2_) Λ CELLCOMPONENT(*x*_2_ , ’*CC*_*Extracellul*a*r* or ’*CC*_*PlasmaMembrane*) Λ UNIFICATIONXREF(*x*_2_ ,*x*_4_) Λ hasOutput(*x*_1_ ,*x*_3_) Λ Entity(*x*_3_) Λ CELLCOMPONENT(*x*_3_, ’*CC*_*Cytosol*) Λ UNIFICATIONXREF(*x*_3_,*x*_5_) Λ sameAs(*x*_4_,*x*_5_)]

It describes that one inputprocess entity should be located in extracellular or plasma membrane; one output entity should be located in cytosol; both entities *x*_2_ and *x*_3_ have the same external reference.

#### Group 5: rules that need cardinality, type, and CELLCOMPONENT property constraints

This group needs a specific type of an entity located in a specific cellular location. The transcription event is of copying information from DNA into new strands of mRNA. The constraints are that the type of the output entity is mRNA with cardinality 1; the location of the output entity is nucleoplasm. The gene expression, ion transport through ion channel, and translation events are included in this group.

Rule for Transcription

*E*: Process(*x*_1_) Λ BIOLOGICALEVENT (*x*_1_, *’ME_ Transcription*)

*C*: ¬ [Ǝ^=1^*x*_2_ hasOutput(*x*_1_,*x*_2_*)* Λ mRNA(*x*_2_) Λ CELLCOMPONENT(*x*_2_, ’*CC_Nucleoplasm)*]

### Criterion 3: validation for systematically correct models

CSO can represent the dynamics of biological pathways and is supposed to simulate complex molecular mechanisms at different levels of details. Once a mathematical model of biological pathways has been generated, it is necessary to estimate free parameters and unknown rate constants on the basis of the experimental data. In this paper, we limit our consideration to generating a simulatable model to be ready for evaluation and focused on protein turnover.

Normally, proteins are synthesized within the cell and over time are gradually broken down into individual amino acids, and this cycle is repeated. To capture this behavior, we define three rules to recognize the entities that are synthesized and degraded. For the entity that is not a product of any process, we add a pre-process that we assume generates the entity. On the other hand, for the entity that will be degraded, a degradation process is added to mimic biological degradation. In the Petri net formalism, adding a pre-process for such entity makes the pre-process to be fired without any constraints when the simulation is started, and the degradation process will consume the entity’s concentration. This complementation of the pathway model in CSO will help users to intuitively understand the given model and the way in which the model works when using Petri net based simulation tools such as Cell Illustrator (CI) [20-22].

In the following rules, the action part improves the given model by adding new instances (*add-instance*) and properties (*add-property*). The variable in braces, e.g. <*x*_2_>, denotes a new instance. Furthermore, the reverse properties are used, e.g. ENTITY^–^(*x*_1_,*x*_4_) is equal to ENTITY(*x*_4_,*x*_1_).

Rule for starting entities

*C*: Entity(*x*_1_) Λ ¬ Complex(*x*_1_) Λ ∀*x*_4_ {ENTITY^–^(*x*_1_,*x*_4_) Λ Input(*x*_4_)}

*A*: *add-instance* Process(<*x*_2_>), OutputProcessBiological(<*x*_3_>)

*add-property* BIOLOGICALEVENT(<*x*_2_>, *’ME_UnknownProduction*), CONNECTOR(<*x*_2_> ,<*x*_3_>), ENTITY(<*x*_3_> ,*x*_1_)

A starting entity is an entity whose type is Entity, but not Complex which is a subclass of Entity, and is connected to a process only via Input connectors. Hence, if a given entity is a starting entity, then the action is to add a unknown production process <*x*_2_> and any necessary properties for it. This rule makes the starting entity be a product of the unknown production process.

Rule for starting complexes

*C*: Complex(*x*_1_) Λ ∀*x*_5_ {ENTITY^–^(*x*_1_,*x*_5_) Λ Input(*x*_5_)}

*A*: *add-instance* Process (<*x*_2_>), OutputProcessBiological(<*x*_4_>)

*add-property *BIOLOGICALEVENT(<*x*_2_>, *’ME_Binding*)*,* CONNECTOR^–^ (<*x*_4_> ,<*x*_2_>)

for ∀*x*_3_ ENTITY(*x*_1_,*x*_3_) Λ Entity(*x*_3_) *do add-property* CONNECTOR^–^ (*x*_3_,<*x*_i_>)

*add-instance* InputProcessBiological(<*x*_i_>)

A starting complex is a starting entity whose type is Complex. For a starting complex, we assume that the complex is generated via a binding process. In the action part, a binding process is added and the components of the complex will be the participants of the binding process.

Rule for degrading entities

*C:* {Protein(*x*_1_) V Complex(*x*_1_) V mRNA(*x*_1_) V SmallMolecule(*x*_1_)} Λ ¬ {Process(*x*_2_) Λ BIOLOGICALEVENT(*x*_2_ , ’*ME_UnknownDegradation*) Λ hasInputProcess(*x*_2_ ,*x*_1_)}

*A*: *add-instance* Process(<*x*_3_>), InputProcessBiological(<*x*_4_>)

*add-property* BIOLOGICALEVENT(<*x*_3_>, *’ME_ UnknownDegradation*), CONNECTOR(<*x*_3_> ,<*x*_4_>)

*add-property* ENTITY(<*x*_4_>,*x*_1_)

For Protein, Complex, mRNA, and SmallMolecule, if a degradation process is not presented, a unknown degradation process is added.

## Results

In order to implement the proposed rule-based system, we used AllegroGraph 3.1 [23] for the CSO data storage and query engine. AllegroGraph is an RDF (Resource Description Framework) graph database with support for SPARQL (SPARQL Protocol and RDF Query Language) [24] as a query language. Query manipulation and CSO data manipulation stored in AllegroGraph are carried out using Protege OWL API [25] and Jena [26]. This system is applied to macrophage models that are manually curated and created by using Cell Illustrator (CI) which is a tool to graphically model and simulate cellular processes.

Scientific publications reflecting the results of biological experiments and including the keywords: Lipopolysaccharide (LPS), phorbol 12-myristate 13-acetate (PMA), macrophage, and signal transduction pathway, were searched from PubMed [27]. A total of 96 publications were selected and modeled by curators. One model was based on a single publication. Basic guidelines on how to create and curate models on CI were provided to the curator. The created model was stored in Cell System Markup Language (CSML) as a default format in CI and exported into CSO.

### Types of warnings

Our validation method was applied to the 96 macrophage models that contained a total of 4910 processes and 7155 entities. The warnings appeared if the expected value in the condition part is not correct or not defined. Table 2 shows the warning description and its frequency in the first and second columns, respectively.

**Table 2:** Description of warnings and their frequencies.

Warning description	Frequency
Criterion 2: validation of biologically correct models	

Cardinality constraint	
1. The number of input/inputprocess/output entities is not correct.	5/18/6
2. The inputprocess/output entities are not defined.	86/2
Type constraint	
3. TYPE of entity is wrong/not defined.	179/16
Property constraint	
4. CELLCOMPONENT is not correct/not given.	657/4
5. FEATURETYPE is not defined.	1361
6. STOICHIOMETRY is not correct.	61
7. UNIFICATIONXREF is not defined.	1501
Property relationship constraint	
8. The values of CELLCOMPONENT that should be different are the same.	87

Criterion 3: validation of systematically correct models	

9. Starting complex that needs to add a binding process.	170
10. Starting entity that needs to add a unknown production process.	3002
11. Degrading entity that needs to add a unknown degradation process.	6885

The macrophage models did not violate the rule for structurally correct models in criterion 1. The reason for this is that the macrophage models were generated by CI, which supports the drawing of Petri net-based models via graphic tools and has the ability to check the connections between processes and entities. Criterion 1 is useful for validating translated data from other databases which have different schemata to CSO like BioPAX2CSO [15,28]. The warnings related to criteria 2 and 3 are given in Table 2.

As described in Methods, the validation rules for criterion 2 generate warnings if a process does not satisfy its constraints. Among of the four constraints in Methods, the cardinality constraint is useful to detect other related problems. If an appropriate entity is not defined, then the related properties of the entity are not satisfied, either. For the property constraint, the FEATURETYPE property is needed for all post-translational modified entities. We found that it was not well guided to curators before curation. The value of this property will be given easily because each rule in group 2 show one-to-one mapping between two properties, BIOLOGICALEVENT and FEATURETYPE. The UNIFICATIONXREF property is used to uniquely identify biological entities. It is important not only for ontology instance data validation, but also for data integration such as model comparison and model merging. Currently, a biological entity is identified by external references that give additional information for the entity. In this work, we use TRANSPATH [29] as a main reference because it provides a comprehensive hierarchy for molecules and distinguishes between different species of the same molecule and between modified and unmodified forms of a protein, which is not supported by other databases [30,31]. For the new molecules to TRANSPATH, especially for modified or complex molecules, it takes time to identify whether the entities have the same basic molecule. We will improve this procedure to reduce the search time.

On the other hand, the rules in criterion 3 directly manipulate the models if the condition part is satisfied. The rules include the action part to complement a given model by adding unknown production processes for starting entities, binding processes for starting complexes, and unknown degradation processes for degrading entities.

From the results, it is useful to analyze the relationship between biological events and warnings to know which points demand careful attention. This feedback is used to give guidelines to curators again.

A total of 44 biological events are used in the 96 macrophage models. Rules are not defined for 8 biological events such as cleavage and unknown interaction, because they have no specific characteristics to distinguish them from others. Such event terms occurred 136 times, which accounts for 2.8% of the processes in the 96 models. As described in Methods, for criterion 2, the 36 rules are divided into the five groups, each of which has same or similar constraints. Table 3 shows the frequency of each biological event occurred in the 96 models, the number of warnings during validation, and the reasons for the warnings. The biological events are listed by the order of rules in the five groups for criterion 2. In the third column, the warnings are counted on the basis of a process and its connected entities. The last column shows the reasons for the warnings per biological event and frequencies in parentheses, except for warnings related to FEATURETYPE and UNIFICATIONXREF properties. For example, *ME_ADPRibosylation* in group 2 occurred two times in the given models. Among the seven warnings, only one was related to the number of input entities.

**Table 3:** Biological event and its frequency used in macrophage models and the number of warnings.

	Biological event	Freq.	Warnings	Reasons (freq.)
1	ME_Autocleavage	2	2	# of input (2)
	ME Binding	1898	253	TYPE (169), # of inputprocess (4), no inputprocess (80)
	ME_DNABinding	29	0	(0)
	ME_DNAReplication	6	0	(0)
	ME_Dissociation	37	3	TYPE (3)
	ME_GDP-GTPExchange	4	0	(0)
	ME_Isomerization	1	0	(0)
	ME_MetabolicReaction	40	7	TYPE (7)
	ME_ProteasomeDegradation	34	0	(0)
	ME_ProteinCleavage	5	0	(0)
	ME_UnknownDegradation	45	1	TYPE (1)

2	ME_Acetylation	3	7	(0)
	ME ADPRibosylation	2	7	# of input (1)
	ME_Amidation	1	1	no output (1)
	ME_Glycosylation	1	3	(0)
	ME_Nitrosylation	2	6	# of inputprocess (1)
	ME Oxidation	12	28	(0)
	ME Phosphorylation	448	967	no inputprocess(1), no output (1)
	ME_Reduction	1	3	(0)
	ME_Sumoylation	2	4	(0)
	ME_Ubiquitination	67	166	(0)
	ME UnknownActivation	793	1415	# of inputprocess (11), no inputprocess (3)
	ME_UnknownInactivation	6	16	(0)
	ME_Autophosphorylation	12	36	# of input (2)
	ME_Dephosphorylation	9	19	(0)
	ME_Deubiquitination	4	11	(0)

3	ME_Dimerization	49	50	STOICHIOMETRY (49),
				# of inputprocess (1)
	ME_Oligomerization	7	7	STOICHIOMETRY (7)
	ME Polymerization	5	5	STOICHIOMETRY (5)

4	ME Internalization	9	22	CELLCOMPONENT (10)
	ME_NuclearExport	4	3	CELLCOMPONENT (3)
	ME Translocation	136	275	# of output (6), no inputprocess (2)
				CELLCOMPONENT (87)

5	ME_GeneExpression	721	262	CELLCOMPONENT (259), TYPE (3)
	ME_IonTransportThroughIonChannel	2	4	TYPE (2)
	ME Transcription	13	7	CELLCOMPONENT (4), TYPE (3)
	ME Translation	364	393	# of input (1), TYPE (7),CELLCOMPONENT(385)

n/a	no rules for 8 biological events	136	-	-

	Total	4910	3983	1121

The biological events are grouped together along with the rules for criteria 2 in Methods. The biological events in the last group have no rules and are notified with n/a in the first column.

### What is corrected by validation?

**Figure 3:**
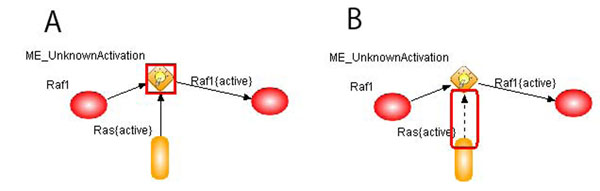
**ME UnknownActivation violating cardinality constraint.** Legend: A and B represent the original model and the corrected model after validation, respectively. The biological event causing warnings and the modified parts are in red boxes in the images. The same legend is used in Figure 4. From the literature, we found that Ras{active} acts as an activator of the process, not as an inputprocess entity. Then, the connector from Ras{active} to the process is changed to a dashed line with an arrow in B.

**Figure 4:**
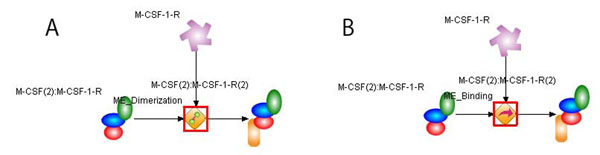
**ME_Dimerization violating constraint and property constraints.** The event needs one inputprocess entity whose stoichiometric coefficient should be equal to 2. We have found that it takes place because of the misassignment of the event term, which is changed into *ME_Binding.*

We checked each model based on the warnings related to the cardinality constraint and corrected each model by reviewing the literature used to generate the model. Two cases are selected to show how our validation approach facilitates to correct the macrophage models. In Figures 3 and 4, A and B indicate the original model and the corrected model after validation, respectively. The red boxes in the figures reveal the places in which the problem happened and the model is changed.

**Case 1:** Misassignment of the connector type. As shown in Figure 3, the *ME_UnknownActivation* event violates the cardinality constraint of inputprocess entities. This event term is used in case the mechanism by which leads to the activation of a molecule is unknown. In the rule for *ME_UnknownActivation*, the condition part describes that only one inputprocess entity is needed to activate. In Figure 3A, we found that there are two inputprocess entities and one of those entities plays a role as an enzyme. Therefore, the type of the connector between the activated Ras and the process is changed into InputAssociationBiological, which represents this event as the activated Ras-induced Raf1 activation as shown in Figure 3B.

**Case 2:** Misassignment of the biological event term. This case shows that one dimerization event also violates the cardinality constraint of inputprocess entities. By the rule, *ME_Dimerization* has only one inputprocess entity whose stoichiometric coefficient is 2. As shown in Figure 4A, the output entity is a complex M-CSF(2):M-CSF-1-R(2) generated by the binding of M-CSF(2):M-CSF-1-R to M-CSF-1-R. We found that the biological event term is assigned mistakenly. Then the term is changed from *ME_Dimerization to ME_Binding* as shown in Figure 4B.

## Discussion and conclusions

To our knowledge, the validation of ontology-based instance data for biological pathways has not been addressed yet. Although the ontology schema is developed with documentation, the use of the ontology is usually separated from the development. The generation of data on the basis of the ontology schema is apt to contain misuse and misunderstanding of the ontology. Such errors are not detected by ontology validation carried out on the basis of the ontology schema. The error correction is usually done manually and is time consuming. As shown in Results, relatively simple rules can detect the errors in the model, such as misassignment and misuse of ontology concepts and terms and enhance the model to be ready for simulation.

Our rule-based validation enables us to provide pathway models that allow computational tools to explore the possible dynamic behavior of pathway components with considering biological meaning. If sophisticated adjustment of quantitative parameters is needed for simulation, the correct assignment of biological concepts and terms are essential for ontology based computational tools. Therefore, this approach can serve as a preprocessing step for model integration, exchange and extraction data, and simulation. In future work, we plan to develop this system as a plug-in for ontology editors, and modeling and simulating tools.

## Competing interests

The authors declare they have no competing interests.

## Authors' contributions

EJ and MN conceived the basic idea. KU and MN created the macrophage models to be evaluated. EJ defined the rules for the validation and implemented the rule-based system. EJ and MN evaluated the validation result and completed the manuscript. SM supervised the whole study. All authors read and approved of the final manuscript.

## Supplementary Material

Additional file 1 The full list of 40 rules. Click here for file
