# Supplementary material for: Ontology-based instance data validation for high-quality curated biological pathways
Source: BMC Bioinformatics. 2011 Feb 15;12(Suppl 1):S8. doi: 10.1186/1471-2105-12-S1-S8 (PMC3044316; doi:10.1186/1471-2105-12-S1-S8)
Supplement: Additional file 1 — The full list of 40 rules. [file 1471-2105-12-S1-S8-S1.pdf]

Additional file 1: The formal description and full list of 40 rules.

A rule is a form of reactive rules, i.e., event-condition-action rules. When the event happens, the corresponding condition is evaluated and the action is executed. Some rules are a form of condition-action rules that directly evaluate the specified conditions with no event. For such rules, if the conditions are satisfied then the action is applied. Relations used in rules are in typewriter type and the details are as follows:

- Unary relations are classes defined in CSO. `Process`(p_1) and `Connector`(c_1) mean that p_1 and c_1 are instances of the `Process` and `Connector` classes, respectively.
- Binary relations in all capital letters are properties defined in CSO, which map instances of a class to instances of another class. `CONNECTOR`(p_1, c_1) means that p_1 and c_1 are related via the `CONNECTOR` property.
- Binary relations in a Hungarian notation, i.e. capital letters for dividing multiple words in a name except the first letter of the name, are user-defined properties by using CSO properties. The `hasInput`(p_1, e_1) property implies that p_1 has an input entity e_1. This kind of relations is needed to define relations among more than three classes. The details will be described later.
- Pre-defined instances in CSO and variables for instances are in italics. For the pre-defined instances in CSO, the apostrophe prefix is used to distinguish it from a variable, such as *'FT_phosphorylated* and *'ME_Binding*.

For the details of CSO and its schema, please refer to [16].

Criterion 1: validation for structurally correct models

Rule 1. If there is given one process and one entity, then there should be only one connector between them. Otherwise, alert.

Event	`Process`(x_1) $\wedge$ `Entity`(x_2)
Condition	$\neg$ [$\exists^{\leqslant 1} x_3$ `CONNECTOR`(x_1,x_3) $\wedge$ {`InputProcessBiological`(x_3) $\vee$ `InputInhibitorBiological`(x_3) $\vee$ `InputAssociationBiological`(x_3) $\vee$ `OutputProcessBiological`(x_3)} $\wedge$ `ENTITY`(x_3,x_2)]
Action	*alert*

Criterion 2: validation for biologically correct models

To represent rules for Criterion 2, we first describe three properties that are not defined in CSO. As described before, we are interested in biological interactions and the related four types of connectors. We define that an input entity is the entity that is connected to a process via one of three input connectors `InputAssociationBiological`, `InputInhibitorBiological`, and `InputProcessBiological`. In particular, the entity connected to a process via `InputProcessBiological` is called the inputprocess entity in order to distinguish it from the other two types of input entities. Lastly, the output entity is defined as the entity connected to a process via `OutputProcessBiological`. Formally, the three properties are as follows:

$\exists x_1, \exists x_2, \exists x_3$ [`hasInput`(x_1,x_3) $\equiv$ `CONNECTOR`(x_1,x_2) $\wedge$ {`InputProcessBiological`(x_2) $\vee$ `InputAssociationBiological`(x_2) $\vee$ `InputInhibitorBiological`(x_2)} $\wedge$ `ENTITY`(x_2,x_3)]

$\exists x_1, \exists x_2, \exists x_3$ [`hasInputProcess`(x_1,x_3) $\equiv$ `CONNECTOR`(x_1,x_2) $\wedge$ `InputProcessBiological`(x_2) $\wedge$ `ENTITY`(x_2,x_3)]

$\exists x_1, \exists x_2, \exists x_3$ [hasOutput(x_1,x_3) $\equiv$ CONNECTOR(x_1,x_2) $\wedge$ OutputProcessBiological(x_2) $\wedge$ ENTITY(x_2,x_3)]

In the rules, `sameAs` defines an equality relationship between two instances or two values, whereas `differentFrom` defines an inequality relationship.

[Group 1: rules that need cardinality and type constraints]

Rule 2. It needs only one input and one output entities, but should not have any regulator entities such as input associate and input inhibitor entities.

Event	Process(x_1) $\wedge$ BIOLOGICALEVENT(x_1, *'ME_Autocleavage*)
Condition	$\neg$ [$\exists^{=1} x_2, \exists^{=1} x_3$ hasInput(x_1,x_2) $\wedge$ hasInputProcess(x_1,x_3)]

Rule 3. It needs at least two inputprocess entities and one output entity whose type is `Complex`.

Event	Process(x_1) $\wedge$ BIOLOGICALEVENT(x_1, *'ME_Binding*)
Condition	$\neg$ [$\exists^{\geqslant 2} x_2, \exists^{\geqslant 1} x_3$ hasInputProcess(x_1,x_2) $\wedge$ Complex(x_2) $\wedge$ hasOutput(x_1,x_3))]

Rule 4. It needs at least two inputprocess entities and one output entity. One inputprocess entity should have a type as `Dna` and one output entity as `Complex`.

Event	Process(x_1) $\wedge$ BIOLOGICALEVENT(x_1, *'ME_DNABinding*)
Condition	$\neg$ [$\exists^{\geqslant 2} x_2, \exists^{\geqslant 1} x_3$ hasInputProcess(x_1,x_2) such that for one of x_2s, Dna(x_2) $\wedge$ hasOutput(x_1,x_3) $\wedge$ Complex(x_3)]

Rule 5. It needs one input entity and one output entity as `Dna`.

Event	Process(x_1) $\wedge$ BIOLOGICALEVENT(x_1, *'ME_DNAReplication*)
Condition	$\neg$ [$\exists^{=1} x_2, \exists^{=1} x_3$ hasInput(x_1,x_2) $\wedge$ Dna(x_2) $\wedge$ hasOutput(x_1,x_3) $\wedge$ Dna(x_3)]

Rule 6. It needs only one inputprocess entity whose type is `Complex` and at least two output entities.

Event	Process(x_1) $\wedge$ BIOLOGICALEVENT(x_1, *'ME_Dissociation*)
Condition	$\neg$ [$\exists^{=1} x_2, \exists^{\geqslant 2} x_3$ hasInputProcess(x_1,x_2) $\wedge$ Complex(x_2) $\wedge$ hasOutput(x_1,x_3)]

Rule 7. It needs at least one inputprocess entity whose name is GTP and one output entity whose name is GDP.

Event	Process(x_1) $\wedge$ BIOLOGICALEVENT(x_1, *'ME_GDPGTPExchange*)
Condition	$\neg$ [$\exists^{\geqslant 1} x_2, \exists^{\geqslant 1} x_3$ hasInputProcess(x_1,x_2) $\wedge$ hasOutput(x_1,x_3) $\wedge$ SmallMolecule(x_2) $\wedge$ NAME(x_2, *'GTP*) $\wedge$ SmallMolecule(x_3) $\wedge$ NAME(x_3, *'GDP*)]

Rule 8. It needs only one inputprocess and at least one output entity both of whose types are `SmallMolecule`.

Event	Process(x_1) $\wedge$ BIOLOGICALEVENT(x_1, *'ME_Isomerization*)
Condition	$\neg$ [$\exists^{=1} x_2, \exists^{\geqslant 1} x_3$ hasInputProcess(x_1,x_2) $\wedge$ SmallMolecule(x_2) $\wedge$ hasOutput(x_1,x_3) $\wedge$ SmallMolecule(x_3)]

Rule 9. It needs at least one inputprocess and at least one output entity both of whose types are SmallMolecule.

Event	Process(x_1) $\wedge$ BIOLOGICALEVENT(x_1, *'ME_MetabolicReaction*)
Condition	$\neg$ [$\exists^{\geqslant 1} x_2, \exists^{\geqslant 1} x_3$ hasInputProcess(x_1,x_2) $\wedge$ SmallMolecule(x_2) $\wedge$ hasOutput(x_1,x_3) $\wedge$ SmallMolecule(x_3)]

Rule 10. It needs only one inputprocess entity whose type is Protein or Complex.

Event	Process(x_1) $\wedge$ BIOLOGICALEVENT(x_1, *'ME_ProteasomeDegradation*)
Condition	$\neg$ [$\exists^{=1} x_2$ hasInputProcess(x_1,x_2) $\wedge$ {Protein(x_2) $\vee$ Complex(x_2)}]

Rule 11. It needs one inputprocess entity whose type is Protein.

Event	Process(x_1) $\wedge$ BIOLOGICALEVENT(x_1, *'ME_ProteinCleavage*)
Condition	$\neg$ [$\exists^{=1} x_2$ hasInputProcess(x_1,x_2) $\wedge$ Protein(x_2)]

Rule 12. It needs one inputprocess entity whose type is Protein, Complex, mRNA, Dna, or SmallMolecule.

Event	Process(x_1) $\wedge$ BIOLOGICALEVENT(x_1, *'ME_UnknownDegradation*)
Condition	$\neg$ [$\exists^{=1} x_2$ hasInputProcess(x_1,x_2) $\wedge$ {Protein(x_2) $\vee$ Complex(x_2) $\vee$ mRNA(x_2) $\vee$ Dna(x_2) $\vee$ SmallMolecule(x_2)}]

[Group 2: cardinality and FEATURETYPE property constraints]

In the following rules, hasFeature(x_1,'x_2) implies that an entity x_1 has a feature type as 'x_2 when 'x_2 is a predefined term for FeatureType in CSO and the formal definition is as follows:

$\exists x_1, \exists x_2, \exists x_3$ [Entity(x_1) $\wedge$ hasFeature(x_1, 'x_2) $\equiv$ SEQUENCEFEATURE(x_1,x_3) $\wedge$ SequenceFeature(x_3) $\wedge$ FEATURETYPE(x_3, 'x_2)) $\wedge$ FeatureType('x_2)]

Note that the instances with prefix (') such as *'ME_Autophosphorylation* and *'FT_phosphorylated* are pre-defined terms (instances) in CSO.

Rules 13–24. It needs at least one inputprocess and one output entities both of whose uni-molecule references (XREF) are same. The output entity should have a feature type which is a pre-defined value.

Event	Process(x_1) $\wedge$ BIOLOGICALEVENT(x_1, *'ME_Acetylation/'ME_ADPRibosylation/ 'ME_Amidation/'ME_Glycosylation/'ME_Nitrosylation/ 'ME_Oxidation/'ME_Phosphorylation/'ME_Reduction/ 'ME_Sumoylation/'ME_Ubiquitination /'ME_UnknownActivation/ 'ME_UnknownInactivation*)
Condition	$\neg$ [$\exists^{=1} x_2, \exists x_3, \exists x_4, \exists x_5$ hasInputProcess(x_1,x_2) $\wedge$ hasOutput(x_1,x_3) $\wedge$ Entity((x_2) $\wedge$ Entity(x_3) $\wedge$ FEATURETYPE(x_3, *'FT_Acetylated/'FT_ADPRibosylated/'FT_Amidated/'FT_Glycosylated/ 'FT_Nitrosylated/'FT_Oxidated/'FT_Phosphorylated/'FT_Reduced/'FT_Sumoylated/ 'FT_Ubiquitinated/ 'FT_Activated/'FT_Inactivated*) $\wedge$ UNIFICATIONXREF(x_2,x_4) $\wedge$ UNIFICATIONXREF(x_3,x_5) $\wedge$ sameAs(x_4,x_5)]

Rule 25. It needs only one inputprocess and one output entities, but should not have any regulator entities such as input associate and input inhibitor entities.

Event $\texttt{Process}(x_1) \wedge \texttt{BIOLOGICALEVENT}(x_1, \textit{'ME\_Autophosphorylation})$
Condition $\neg\ [\exists^{=1}x_2, \exists x_3, \exists x_4, \exists x_5, \exists^{=1}x_6\ \texttt{hasInputProcess}(x_1,x_2) \wedge \texttt{hasOutput}(x_1,x_3) \wedge \texttt{Entity}(x_2) \wedge \texttt{Entity}(x_3) \wedge \texttt{FEATURETYPE}(x_3, \textit{'FT\_Autophosphorylated}) \wedge \texttt{UNIFICATIONXREF}(x_2,x_4) \wedge \texttt{UNIFICATIONXREF}(x_3,x_5) \wedge \texttt{sameAs}(x_4,x_5) \wedge \texttt{hasInput}(x_1,x_6) \wedge \texttt{Entity}(x_6)]$

Rules 26–27. It nees only one inputprocess entity and at least one output entity. The inputprocess entity should have a defined feature type. For example, a phosphorylated entity can be dephosphorylated. So the inputprocess entity has a feature type as *'FT_phosphorylated.*

Event $\texttt{Process}(x_1) \wedge \texttt{BIOLOGICALEVENT}(x_1, \textit{'ME\_Dephosphorylation/'ME\_Deubiquitination})$
Condition $\neg\ [\exists^{=1}x_2, \exists^{\geqslant 1}x_3, \exists x_4, \exists x_5\ \texttt{hasInputProcess}(x_1,x_2) \wedge \texttt{FEATURETYPE}(x_2, \textit{'FT\_phosphorylated/'FT\_ubiquitinated}) \wedge \texttt{hasOutput}(x_1,x_3) \wedge \texttt{UNIFICATIONXREF}(x_2,x_4) \wedge \texttt{UNIFICATIONXREF}(x_3,x_5) \wedge \texttt{sameAs}(x_4,x_5)]$

[Group 3: cardinality and STOICHIOMETRY property constraints]

In CSO, the stoichiometric coefficient is the property of the connector connecting one process and one inputprocess entity, because the same entity can be involved in many processes and the stoichiometric coefficient will be different depending on the involved processes. In these rules, `hasStoichiometry` implies that given a process x_1, the participating inputprocess entity x_3 has x_7 as its stoichiometric coefficient.

$$\exists x_1, \exists x_2, \exists x_3, \exists x_4, \exists x_5, \exists x_6, \exists x_7\ [\texttt{hasInputProcess}(x_1,x_3) \wedge \texttt{hasStoichiometry}(x_3,x_7) \equiv \texttt{CONNECTOR}(x_1,x_2) \wedge \texttt{InputProcessBiological}(x_2) \wedge \texttt{ENTITY}(x_2,x_3) \wedge \texttt{CONNECTORSIMULATIONPROPERTY}(x_2,x_4) \wedge \texttt{ConnectorSimulationProperty}(x_4) \wedge \texttt{CONNECTORKINETIC}(x_4,x_5) \wedge \texttt{ConnectorKinetic}(x_5) \wedge \texttt{PARAMETER}(x_5,x_6) \wedge \texttt{Parameter}(x_6) \wedge \texttt{KEY}(x_6, \textit{'stoichiometry}) \wedge \texttt{VALUE}(x_6,x_7)]$$

Rule 28. It needs only one inputprocess entity whose stoichiometry coefficient is equal to 2 and only one output entity whose type is `Complex`.

Event $\texttt{Process}(x_1) \wedge \texttt{BIOLOGICALEVENT}(x_1, \textit{'ME\_Dimerization})$
Condition $\neg\ [\exists^{=1}x_2, \exists^{=1}x_3, \exists^{=1}x_4\ \texttt{hasInputProcess}(x_1,x_2) \wedge \texttt{Entity}(x_2) \wedge \texttt{hasStoichiometry}(x_2,x_3) \wedge (x_3 = 2) \wedge \texttt{hasOutput}(x_1,x_4) \wedge \texttt{Complex}(x_4)]$

Rule 29. It needs only one inputprocess entity whose stoichiometry coefficient is from 3 to 20 and only one output entity whose type is `Complex`.

Event $\texttt{Process}(x_1) \wedge \texttt{BIOLOGICALEVENT}(x_1, \textit{'ME\_Oligomerization})$
Condition $\neg\ [\exists^{=1}x_2, \exists^{=1}x_3, \exists^{=1}x_4\ \texttt{hasInputProcess}(x_1,x_2) \wedge \texttt{Entity}(x_2) \wedge \texttt{hasStoichiometry}(x_2,x_3) \wedge (3 \leqslant x_3 \leqslant 20) \wedge \texttt{hasOutput}(x_1,x_4) \wedge \texttt{Complex}(x_4)]$

Rule 30. It needs only one inputprocess entity whose stoichiometry coefficient is 21 or more and only one output entity whose type is `Complex`.

Event Process(x_1) $\wedge$ BIOLOGICALEVENT(x_1, *'ME_Polymerization*)
Condition $\neg$ [$\exists^{=1}x_2, \exists^{=1}x_3, \exists^{=1}x_4$ hasInputProcess(x_1,x_2) $\wedge$ Entity(x_2) $\wedge$ hasStoichiometry(x_2,x_3) $\wedge$ ($x_3 \geqslant 21$) $\wedge$ hasOutput(x_1,x_4) $\wedge$ Complex(x_4)]

[Group 4: cardinality and CELLCOMPONENT property constraints]

Rule 31. It needs only one inputprocess entity whose location is extracellular or plasma membrane, and only one output entity whose location is cytoplasm.

Event Process(x_1) $\wedge$ BIOLOGICALEVENT(x_1, *'ME_Internalization*)
Condition $\neg$ [$\exists^{=1}x_2, \exists^{=1}x_3, \exists x_4, \exists x_5$ hasInputProcess(x_1,x_2) $\wedge$ Entity(x_2) $\wedge$ CELLCOMPONENT(x_2, *'CC_Extracellular* or *'CC_PlasmaMembrane*) $\wedge$ UNIFICATIONXREF(x_2,x_4) $\wedge$ hasOutput(x_1,x_3) $\wedge$ Entity(x_3) $\wedge$ CELLCOMPONENT(x_3, *'CC_Cytoplasm*) $\wedge$ UNIFICATIONXREF(x_3,x_5) $\wedge$ sameAs(x_4,x_5)]

Rule 32. It needs only one inputprocess entity whose location is nucleoplasm and only one output entity whose location is cytoplasm.

Event Process(x_1) $\wedge$ BIOLOGICALEVENT(x_1, *'ME_NuclearExport*)
Condition $\neg$ [$\exists^{=1}x_2, \exists^{=1}x_3$ hasInputProcess(x_1,x_2) $\wedge$ Entity(x_2) $\wedge$ CELLCOMPONENT(x_2, *'CC_Nucleoplasm*) $\wedge$ hasOutput(x_1,x_3) $\wedge$ Entity((x_3) $\wedge$ CELLCOMPONENT(x_3, *'CC_Cytoplasm*)]

Rule 33. It needs only one inputprocess and only one output entities. In addition, the location of two entities should be different.

Event Process(x_1) $\wedge$ BIOLOGICALEVENT(x_1, *'ME_Translocation*)
Condition $\neg$ [$\exists^{=1}x_2, \exists^{=1}x_3, \exists x_4, \exists x_5, \exists x_6, \exists x_7$ hasInputProcess(x_1,x_2) $\wedge$ hasOuputProcess(x_1,x_3) $\wedge$ CELLCOMPONENT(x_2,x_4) $\wedge$ CELLCOMPONENT(x_3,x_5) $\wedge$ differentFrom(x_4,x_5) $\wedge$ UNIFICATIONXREF(x_2,x_6) $\wedge$ UNIFICATIONXREF(x_3,x_7) $\wedge$ sameAs(x_6,x_7)]

[Group 5: cardinality, type, and CELLCOMPONENT property constraints]

Rule 34. It needs only one output entity whose type is mRNA and whose location is nucleoplasm.

Event Process(x_1) $\wedge$ BIOLOGICALEVENT(x_1, *'ME_GeneExpression*)
Condition $\neg$ [$\exists^{=1}x_2$ hasOutput(x_1,x_2) $\wedge$ mRNA(x_2) $\wedge$ CELLCOMPONENT(x_2, *'CC_Nucleoplasm*)]

Rule 35. It needs only one inputprocess and only one output entities both of whose types are SmallMolecule, but the location of two entities should be different.

Event Process(x_1) $\wedge$ BIOLOGICALEVENT(x_1, *'ME_IonTransportThroughIonChannel*)
Condition $\neg$ [$\exists^{=1}x_2, \exists^{=1}x_3, \exists x_4, \exists x_5, \exists x_6, \exists x_7$ hasInputProcess(x_1,x_2) $\wedge$ SmallMolecule(x_2) $\wedge$ CELLCOMPONENT(x_2,x_4) $\wedge$ UNIFICATIONXREF(x_2,x_6) $\wedge$ hasOutput(x_1,x_3) $\wedge$ SmallMolecule(x_3) $\wedge$ CELLCOMPONENT(x_3,x_5) $\wedge$ differentFrom(x_4,x_5) $\wedge$ UNIFICATIONXREF(x_3,x_7) $\wedge$ sameAs(x_5,x_7)]

Rule 36. It needs only one output entity whose location is nucleoplasm and whose type is mRNA.

Event $\texttt{Process}(x_1) \wedge \texttt{BIOLOGICALEVENT}(x_1, \textit{'ME\_Transcription})$
Condition $\neg\ [\exists^{=1} x_2\ \texttt{hasOutput}(x_1, x_2) \wedge \texttt{mRNA}(x_2) \wedge \texttt{CELLCOMPONENT}(x_2, \textit{'CC\_Nucleoplasm})]$

Rule 37. It needs only one input entity whose type is `mRNA` located in nucleoplasm, and only one output entity whose type is `Protein` located in cytoplasm.

Event $\texttt{Process}(x_1) \wedge \texttt{BIOLOGICALEVENT}(x_1, \textit{'ME\_Translation})$
Condition $\neg\ [\exists^{=1} x_2, \exists^{=1} x_3\ \texttt{hasInput}(x_1, x_2) \wedge \texttt{Entity}(x_2) \wedge$
$\texttt{CELLCOMPONENT}(x_2, \textit{'CC\_Nucleoplasm}) \wedge \texttt{hasOutput}(x_1, x_3) \wedge \texttt{Protein}(x_3)$
$\wedge\ \texttt{CELLCOMPONENT}(x_3, \textit{'CC\_Cytoplasm})]$

Criterion 3: validation for systematically correct models

In the following three rules, the action complements the given model by adding new instances (*add-instance*) and properties (*add-property*). The variable in braces, e.g. $\{\texttt{x}_2\}$, denotes a new instance ID. Furthermore, the reverse properties are used, e.g. $\texttt{ENTITY}^{-}(x_1,x_4)$ is equal to $\texttt{ENTITY}(x_4,x_1)$.

Rule 38. % Rule for starting entities

Condition $\texttt{Entity}(x_1) \wedge \neg\ \texttt{Complex}(x_1) \wedge \forall x_4\ \{\texttt{ENTITY}^{-}(x_1, x_4) \wedge \texttt{Input}(x_4)\}$
Action
add-instance $\texttt{Process}(<x_2>)$, $\texttt{OutputProcessBiological}(<x_3>)$
add-property $\texttt{BIOLOGICALEVENT}(<x_2>, \textit{'ME\_UnknownProduction})$,
$\texttt{CONNECTOR}(<x_2>, <x_3>)$, $\texttt{ENTITY}(<x_3>, x_1)$

Rule 39. % Rule for starting complexes

Condition $\texttt{Complex}(x_1) \wedge \forall x_5 \{\texttt{ENTITY}^{-}(x_1, x_5) \wedge \texttt{Input}(x_5)\}$
Action
add-instance $\texttt{Process}(<x_2>)$, $\texttt{OutputProcessBiological}(<x_4>)$
add-property $\texttt{BIOLOGICALEVENT}(<x_2>, \textit{'ME\_Binding})$,
$\texttt{CONNECTOR}^{-}(<x_4>, <x_2>)$
for $\forall x_3\ \texttt{ENTITY}(x_1, x_3) \wedge \texttt{Entity}(x_3)$ *do*
add-property $\texttt{CONNECTOR}^{-}(x_3, <x_i>)$
add-instance $\texttt{InputProcessBiological}(<x_i>)$

Rule 40. % Rule for degrading entities

Condition $\{\texttt{Protein}(x_1) \vee \texttt{Complex}(x_1) \vee \texttt{mRNA}(x_1) \vee \texttt{SmallMolecule}(x_1)\} \wedge$
$\neg\ \{\texttt{Process}(x_2) \wedge \texttt{BIOLOGICALEVENT}(x_2, \textit{'ME\_UnknownDegradation}) \wedge$
$\texttt{hasInputProcess}(x_2, x_1)\}$
Action
add-instance $\texttt{Process}(<x_3>)$, $\texttt{InputProcessBiological}(<x_4>)$
add-property $\texttt{BIOLOGICALEVENT}(<x_3>, \textit{'ME\_UnknownDegradation})$,
$\texttt{CONNECTOR}(<x_3>, <x_4>)$, $\texttt{ENTITY}(<x_4>, x_1)$
